# Protective action of taurine, given as a pretreatment or as a posttreatment, against endotoxin-induced acute lung inflammation in hamsters

**DOI:** 10.1186/1423-0127-17-S1-S19

**Published:** 2010-08-24

**Authors:** Tapan M Bhavsar, Sanket N Patel, Cesar A Lau-Cam

**Affiliations:** 1 Department of Pharmaceutical Sciences, St. John’s University, College of Pharmacy and Allied Health Professions, 8000 Utopia Parkway, Jamaica, New York 11439, USA

## Abstract

To assess the effect of taurine on lipopolysaccharide (LPS)-induced lung inflammation, oxidative stress and apoptosis, female Golden Syrian hamsters were intratracheally instilled with bacterial LPS (0.02 mg in phosphate buffered saline (PBS) pH 7.4), before or after a 3-day intraperitoneal treatment with a single dose of taurine (50 mg/kg/day in PBS pH 7.4), and bronchoalveolar lavage fluid (BALF) and lung tissue samples were collected at 24 hr after the last treatment. In comparison to BALF samples from animals receiving only PBS pH 7.4, and serving as controls, those of LPS-stimulated animals exhibited a higher count of both total leukocytes and neutrophils and increased expression of tumor necrosis factor receptor 1. In comparison to lungs from control animals, those from LPS-treated animals showed increased cellular apoptosis, lipid peroxidation, decreased glutathione levels, altered activities of antioxidant enzymes (catalase, glutathione peroxidase, superoxide dismutase) and focal inflammation confined to the parenchyma. A treatment with taurine was found to significantly attenuate all these alterations, with the protection being, in all instances, greater when given before rather than after LPS. The present results suggest that taurine is endowed with antiinflammatory and antioxidant properties that are protective in the lung against the deleterious actions of Gram negative bacterial endotoxin.

## Background

Acute lung injury (ALI) is a characteristic sequel to infection by Gram negative bacteria and an important cause of morbidity and mortality in humans [1]. A common causative factor of ALI is lipopolysaccharide (LPS), an endotoxin present in the bacterial outer membrane [2]. Typical manifestations of ALI are alveolar and airway inflammatory response [3,4], the presence of inflammatory cells and proteinaceous fluid in air spaces [5,6], increased microvascular permeability due to endothelial barrier disruption [7,8], bronchoalveolar cell death [9], and cellular changes suggestive of lung inflammation and/or injury [10]. One major contributory factor to the pathogenesis of ALI is the release of reactive oxygen species (ROS) and reactive nitrogen species (RNS), proteolytic enzymes, lipid mediators and proinflammatory cytokines principally from neutrophils and alveolar and interstitial macrophages [9]. The ensuing overwhelming oxidative and nitrosative stresses [10,11], in turn, cause direct damage to DNA [9], apoptosis [12], deplete reduced glutathione (GSH) stores [13-15], promote lipid peroxidation (LPO)[16,17], protein nitration and protein activity alteration [18,19], inactivate antioxidant and antiproteinase enzymes [9], and activate transcriptional factors mediating the expression of proinflammatory genes in phagocytic cells and in endothelial and epithelial lung cells [9,20-22].

The relevance of oxidative stress to the development of ALI is supported by the results of studies in experimental animal models of ALI demonstrating that low molecular weight antioxidant compounds possessing a wide range of structural features and biological activities are able to decrease the severity of the inflammatory process by reducing the migration of macrophages, monocytes and neutrophils into the lung [23] and the production of ROS and RNS by these cells [24,25]. One of the compounds that has demonstrated protective actions in the lung against inflammation by LPS and other exogenous agents is taurine (TAU), a nonprotein amino acid with a ubiquitous distribution and a high concentration in human tissues. As an antioxidant, TAU is rather unique since it is able to attenuate LPO and the loss of intracellular antioxidant defenses under conditions of oxidative stress in spite of lacking a readily oxidizable functionality [26] and has the ability to selectively scavenge free radicals generated during ALI [27,28]. For example, the addition of this compound to cultured pneumocytes was found to reduce the LPS-induced generation of ROS and the activation of mitogen-activated protein kinases and Bax [29]; and the pretreatment of rats with 5% TAU in the drinking water resulted in a lower number of inflammatory leukocytes infiltrating the lung and in attenuation of the focal bronchiolar hyperplasia that developed from a short contact with ozone [30]. Moreover, an earlier study from this laboratory determined that a 3-day treatment of hamsters with TAU was able to reduce the number of proinflammatory leukocytes, the expression of tumor necrosis factor receptor 1 (TNFR1) on macrophages, the activation of caspase-3 activity and accompanying apoptosis, LPO and the decreases in GSH and activities of antioxidant enzymes in bronchoalveolar lavage fluid (BALF) samples as a result of a challenge with LPS [31]. On the basis of these results, the present study was undertaken in hamsters with the specific purpose of determining: (a) the effects of TAU on the inflammation, oxidative stress and apoptosis that develops in lung tissue as a result of an exposure to LPS, (b) the role of the order of administration of TAU relative to that of LPS on the magnitude of the effects demonstrated by TAU, and (c) the extent to which the findings for lung tissue samples correlate with those gathered for markers of inflammation in BALF samples.

## Methods

### Materials and chemicals

All the chemicals, reagents and assay kits used in the study were purchased from commercial sources in the USA. H_2_O_2_ (30% w/w), LPS (serotype: O26:B6 obtained from American Type Culture Collection no. 12795; with short chain-length approximating that of mutant rough strain LPS), PBS, GSH, TAU, TCA, SSA, TEP and TBA were from Sigma-Aldrich, St. Louis, MO; 0.1 N and 6 N HCl were from Mallinckrodt Baker, Inc., Phillipsburg, NJ; and metaphosphoric acid was from Aldrich Chemical Co., Inc., Milwaukee, WI.

### Animals

Female Golden Syrian hamsters (5-6 weeks old, 100±15 g in weight, 6 per group) were purchased from Harlan, Indianapolis, IN, USA. The animals were housed in a temperature-controlled room (21±1°C) with a 12 hr light-12 hr dark cycle; and had free access to a standard hamster chow and filtered tap water for at least 7 days. The study received the approval of the Institutional Animal Care and Use Committee of St. John’s University, and the animals were cared in accordance with the guidelines established by the United States Department of Agriculture.

### Treatments with LPS and TAU

To determine the effects of a pretreatment with taurine on LPS-induced lung injury, hamsters were treated with TAU (as a solution in phosphate buffered saline (PBS) pH 7.4, 50 mg/kg/0.5 ml/day) by the intraperitoneal (i.p.) route for 3 days, followed successively by an i.p. dose of pentobarbital sodium (90 mg/kg/0.4 ml) to induce anesthesia, and an intratracheal (i.t.) instillation of LPS (0.2 ml of 0.1 mg/ml in PBS pH 7.4) on day 4. Controls were treated with: (a) i.p. PBS pH 7.4 for 3 days followed by i.t. LPS on day 4 (positive control), and (b) only i.t. PBS pH 7.4 on day 4 (negative control). To determine the effects of a posttreatment with TAU on LPS-induced lung injury, the hamsters received LPS (0.2 ml of 0.1 mg/ml in PBS pH 7.4) by i.t. instillation on day 1, followed by TAU (as a solution in phosphate buffered saline (PBS) pH 7.4, 50 mg/kg/0.5 ml/day) by i.p. route for 3 days. Control animals were treated with (a) i.t. LPS on day 1 followed by i.p. PBS pH 7.4 on days 2 to 4 (positive control), and (b) only with i.t. PBS pH 7.4 (negative control). All i.t. instillations were carried out using a 1-ml syringe fitted with a 27-gauge needle. Following an i.t. delivery, the incision was closed with metal clips.

### Collection of lung and BALF samples

On day 5, 24 h after a LPS instillation or a TAU treatment, the animals were sacrificed using a high dose of pentobarbital sodium (240 mg/kg/0.7 ml, i.p.), and BALF samples were collected by rinsing the bronchoalveolar surface with PBS pH 7.4, and bringing the volume of the pooled washings to 10 ml with additional PBS pH 7.4. Immediately thereafter, the lungs were surgically removed, washed without delay with ice-cold physiologic saline, patted dry with filter paper, frozen in liquid nitrogen, and kept at -20°C until used in an assay.

### Preparation of lung homogenates

Following their removal, the lung samples were rinsed immediately with physiological saline, patted dry with filter paper, weighed, and perfused with ice-cold physiologic saline. A portion of lung sample was mixed with PBS pH 7.4 in a 1:30 (w/v) ratio and made into a fine homogenate with a hand held tissue homogenizer (Tissue-Tearor^®^, BioSpec Products, Inc., Bartlesville, OK) while keeping the mixture cold with the help of an ice bath. After a short sonication, the suspension was centrifuged at 14,000 rpm for 30 min, and the supernatant used for the assays of MDA, GSH, CAT, SOD and GPx.

### Assay of MDA

The concentration of MDA in the lung was measured as TBARS using the method of Buege and Aust [32]. An aliquot of lung homogenate was mixed with a reagent containing 15% TCA (w/v)-0.375% TBA (w/v)-0.25 N HCl, and the mixture heated at 90^o^C for 1 hr. After allowing the mixture to cool to room temperature, and a brief centrifugation step to remove insolubles, the absorbance of the clear supernatant was read on a spectrophotometer at 535 nm. The concentration of MDA was derived from a standard curve prepared from serial dilutions of a 3.2 µM stock solution of TEP that were treated in identical manner as the lung homogenate samples. The concentration of MDA was expressed as nmol/mg of protein.

### Assay of GSH

The concentration of GSH in the lung homogenate was measured following its reaction with DTNB according to Ellman [33]. An aliquot of lung homogenate was mixed with 5% metaphosphoric acid, the mixture centrifuged at 2000 x g for 5 min, and an aliquot of the clear supernatant was mixed with 1.9 ml of 0.1 M phosphate buffer pH 8.0 and 20 µl of 0.02 M DTNB in 0.1 M phosphate buffer pH 8.0, and the absorbance of the resulting product read at 412 nm on a spectrophotometer. The concentration of GSH in the sample was derived by reference to a calibration curve of GSH prepared from serial dilutions of a 240 µM GSH stock solution that were treated in identical manner as the lung homogenate samples. The result was reported as μmol/mg of protein.

### Assay of CAT activity

The activity of CAT was measured spectrophotometrically as described by Aebi [34]. An aliquot of lung homogenate was mixed with 10 times its volume of PBS pH 7.4 and of 30 mM H_2_O_2_ in a spectrophotometric quartz cuvette, and the absorbance of the reaction mixture read without delay at 240 nm twice, immediately after mixing and 1 min later. The activity of CAT, in U/min/mg of protein, was calculated from the equation [ΔOD•V_c_•df/0.071•V_s_], where ΔOD is the difference between the first and second absorbance readings; V_c_ is the volume of the spectrophotometric cuvette in ml; V_s_ is the volume of sample taken in ml; df is the dilution factor; and 0.071 is the molar extinction coefficient of H_2_O_2_.

### Assay of GPx activity

The activity of GPx was measured indirectly by a coupled reaction with glutathione reductase using the spectrophotometric method of Gϋnzler and Flohé [35]. The reaction mixture contained an aliquot of lung homogenate, glutathione reductase solution (54 U/ml), 10 mM GSH, and 15 mM β-NADPH in PBS pH 8.0. After standing at room temperature, the reaction mixture was mixed with 3 mM H_2_O_2_, and the change in absorbance of the reaction mixture at 340 nm was measured for 1 min. The results are expressed in U/min/mg of lung.

### Assay of SOD activity

This enzyme activity was determined based on the inhibitory action of SOD on the reduction of NBT by superoxide anion generated by a xanthine-xanthine oxidase system and the conditions described by Ukeda et al. [36]. For this purpose, an aliquot of lung homogenate was mixed with 15% bovine serum albumin in PBS pH 8.0, 3 mM xanthine, 3 mM EDTA, 0.75 mM NBT, and 56 U/ml of xanthine oxidase. After allowing the reaction mixture to stand at room temperature for 30 min, its absorbance was read on a spectrophotometer at 560 nm. The enzyme activity was expressed as U/min/mg of lung.

### Histological studies

Following euthanasia of the animals with a high dose of pentobarbital (240 mg/kg/0.7 ml, i.p.), the lungs were fixed *in situ* with 10% neutral-buffered formalin for about 2 hr at a pressure of 20 mm of water. The lungs, along with the attached heart, were surgically removed through a vertical incision along the thorax, and fixed in 10% neutral-buffered formalin for an additional 48 hr. After excising any extrapulmonary structures with the help of a scalpel, the lungs were cut into pieces of a size suitable for histological processing, put though a standard embedding procedure in paraffin, sectioned on a microtome, and stained with H & E. The sections where then examined for evidence of inflammation with the help of a light microscope.

### Determination of lung injury

The presence of lung injury in the tissue sections was graded using the scale described by Szarka et al. [37]. The following scoring values were used: 0 = no reaction in the alveolar walls, 1 = diffuse reaction in the alveolar walls but without thickening of the interstitium, 2 = diffuse presence of inflammatory cells in the alveolar walls with a slight thickening of the interstitium, 3 = moderate interstitial thickening accompanied by inflammatory cell infiltrates, and 4 = interstitial thickening involving more than one-half of the microscopic field. Results were expressed as the average of the values from 50 microscopic fields.

### Immunocytochemistry of TNFR1

Cytocentrifuged lung preparations were fixed with ethanol and permeated with an alcohol-acetic acid (2:1) mixture. Endogenous peroxidase activity was quenched with 0.3% H_2_O_2_ and nonspecific binding was blocked with goat serum (Vector Laboratories, Inc., Burlingame, CA). After an incubation with anti-mouse TNFR1 polyclonal antibody (Stressgen, Victoria, BC, Canada) at room temperature for 30 min, and a rinsing with PBS pH 7.4, the sample was successively incubated with biotinylated goat anti-rabbit antibody or rabbit anti-rat antibody (Stressgen, Victoria, BC, Canada) at room temperature for 1 hr, and with ABC reagent (Vector Laboratories, Inc., Burlingame, VT) for 1 hr, prior to staining with 3,3’-diaminobenzidine substrate in the dark, and counterstaining with methyl green. The slides were examined under a light microscope at 400x magnification for the presence of macrophages stained in brown. For comparative purposes, a negative control sample that had not been incubated with TNFR1 primary antiserum was also examined. The results were expressed as the number of macrophages staining for TNFR1 in a group of 200 cells counted in the same section. The number of macrophages staining for TNFR1 was reported as a percentage of the total number of macrophages examined.

### TUNEL staining for apoptosis

Lung tissue sections were digested with 20 μg/ml of proteinase K (Sigma-Aldrich, St. Louis, MO) at room temperature, washed with distilled water, and treated with 0.3% H_2_O_2_ in PBS pH 7.4 to quench endogenous peroxidase. After incubation with TdT enzyme (Chemicon International, Temecula, CA) at 37°C for 1 hr, the samples were exposed to anti-digoxigenin conjugate (Chemicon International, Temecula, CA) at room temperature for 30 min. The samples were stained with DAB peroxidase substrate (Vector Laboratories, Inc., Burlingame, VT), counterstained with methyl green, and examined under a microscope. Twenty-five high-power (HPF, 400x magnification) microscopic fields were examined, and the results were expressed as the mean number of TUNEL-positive cells per HPF.

### Statistical analysis

The experimental results are expressed as mean ± standard error of the mean (S.E.M.) for n = 6. A significant difference between control and treatment groups was determined by Student’s t-test followed by one-way analysis of variance (ANOVA) and Newman-Keuls multiple-range test. A P value ≤0.05 was taken as an indication of a statistically significant difference.

## Results

### Effects of LPS and TAU-LPS on indices of lung oxidative stress

**Figure 1:**
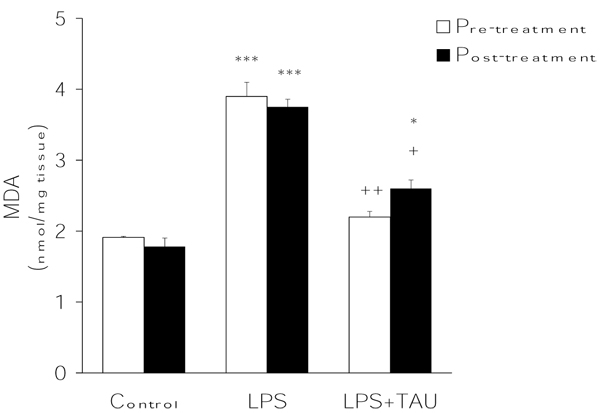
**TAU attenuated LPS-induced formation of MDA in the lung when given before or after LPS.** Each bar represents the mean ± S.E.M. for n = 6. *P<0.05 and ***P<0.001 vs. control; ^+^P<0.05 and ^++^P<0.01 vs. LPS.

Evidence of the occurrence of oxidative stress in the lung as a result of an acute exposure to LPS was inferred from the levels of MDA (as TBARS) and GSH and from the activities of the antioxidant enzymes CAT, GPx and SOD in lung homogenates. As shown in Figure 1, LPS stimulated LPO in lung tissue since it markedly and significantly increased the formation of MDA (by >100%, P<0.001 vs. control). The administration of TAU for 3 days, either before or after LPS, resulted, in both instances, in a significant reduction (by 46%, P<0.01 and 31%, P<0.05, respectively) in MDA formation induced by LPS.

**Figure 2:**
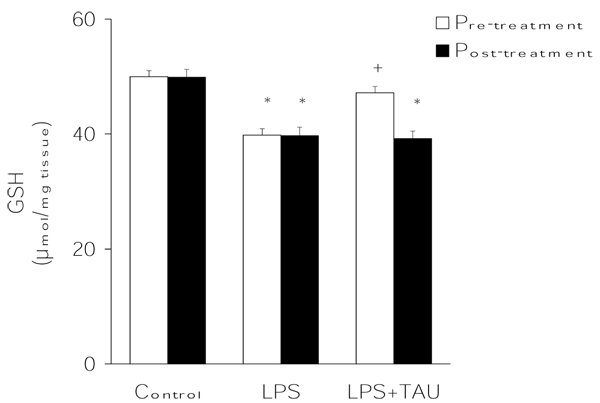
**TAU prevented the LPS-induced depletion of lung GSH when given before, but not after, LPS.** Each bar represents the mean ± S.E.M. for n = 6. ^+^P<0.05 vs. control; ^+^P<0.05 vs. LPS.

The results presented in Figure 2 indicate that, in comparison to control values, LPS was able to reduce the lung GSH to a significant extent (by ≥20%, P<0.05). However, in the presence of TAU, diverging results were obtained depending on the timing of TAU administration. While a pretreatment with this amino acid virtually abolished the effect of LPS on lung GSH (only 6% decrease), a post-treatment was without an obvious effect.

**Figure 3:**
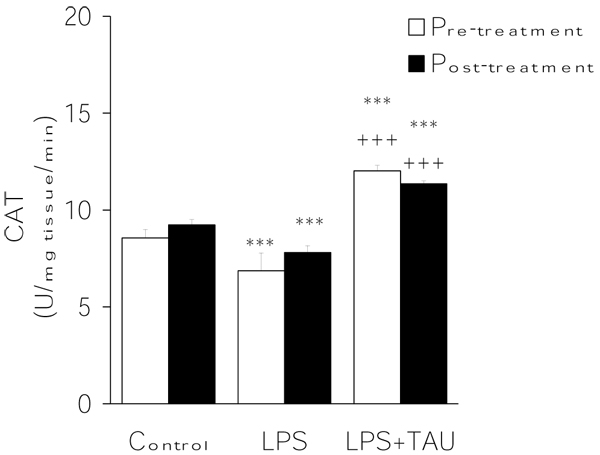
**TAU attenuated the LPS-induced decrease in lung CAT activity when given before or after LPS.** Each bar represents the mean ± S.E.M. for n = 6. ***P<0.001 vs. control; ^+++^P<0.001 vs. LPS.

**Figure 4:**
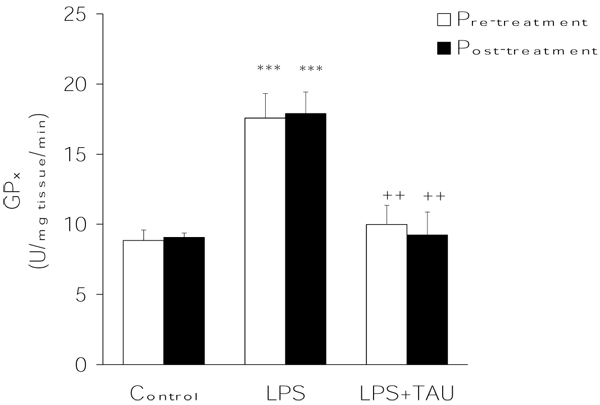
**TAU attenuated the LPS-induced increase in lung GPx activity when given before or after LPS.** Each bar represents the mean ± S.E.M. for n = 6. ***P<0.001 vs. control; ^++^P<0.01 vs. LPS.

**Figure 5:**
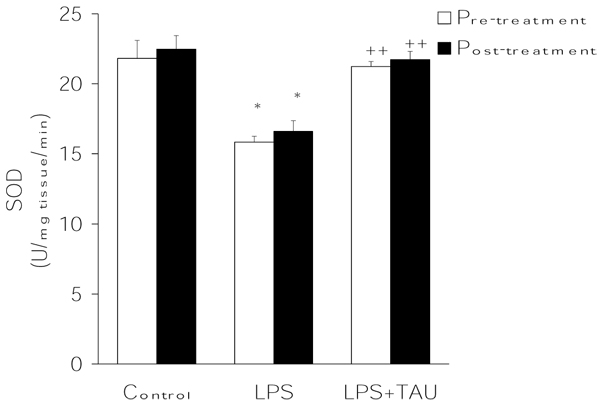
**TAU attenuated the LPS-induced decrease in lung SOD activity when given before or after LPS.** Each bar represents the mean ± S.E.M. for n = 6. *P<0.05 vs. control; ^++^P<0.01 vs. LPS.

From the results summarized in Figures 3,4,5, it is evident that LPS exerted contrasting effects on the activities of the major antioxidant enzymes. On the one hand, it lowered the mean activity values of both CAT (by ~15%, P<0.05) (Figure 3) and SOD (by 27%, P<0.05) (Figure 4) and elevated that of GPX (by ~98%, P<0.001) (Figure 4) in comparison to the respective control values. Regardless of its order of administration relative to that of LPS, TAU was able to counteract these alterations throughout. Thus, in the case of CAT the activities were 75% and ~46% greater than control when given along with LPS as a pretreatment and posttreatment, respectively (both at P<0.001 vs. LPS). Likewise, TAU lowered the increase in SOD activity induced by LPS by about the same extent when given before (31% reduction) or after (34% reduction) LPS (both at P<0.01 vs. LPS). In contrast, while TAU attenuated the elevation in GPx activity caused by LPS, it was somewhat more effective when given as a posttreatment (by ~49%) than as a pretreatment (by 43%) to LPS (both at P<0.01 vs. LPS) (Figure 5).

### Effects of LPS and TAU-LPS on total leukocyte, neutrophil and total neutrophil counts in BALF

**Figure 6:**
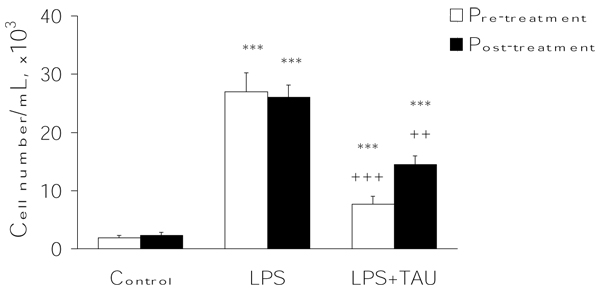
**TAU attenuated the LPS-induced influx of total leukocytes into BALF when given before or after LPS.** Each bar represents the mean ± S.E.M. for n = 6. ***P<0.001 vs. control; ^++^P<0.01 and ^+++^P<0.001 vs. LPS.

**Figure 7:**
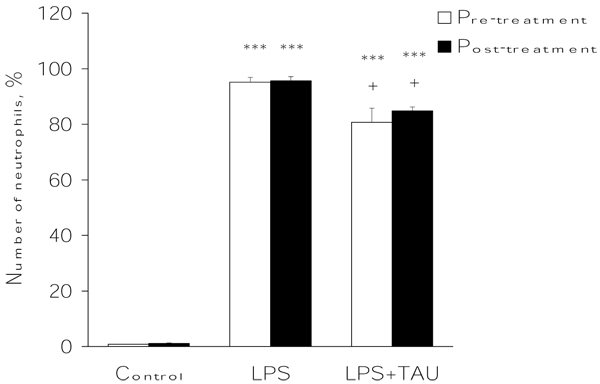
**TAU attenuated the LPS-induced influx of neutrophils into BALF when given before or after LPS.** Each bar represents the mean ± S.E.M. for n = 6. ***P<0.001 vs. control; ^+^P<0.05 vs. LPS.

**Figure 8:**
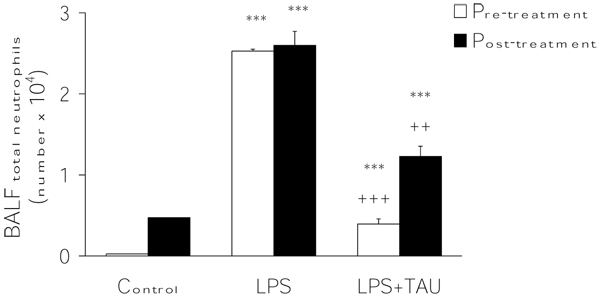
**TAU attenuated the LPS-induced influx of total neutrophils into BALF when given before or after LPS.** Each bar represents the mean ± S.E.M. for n = 6. ***P<0.001 vs. control; ^++^P<0.01 and ^+++^P<0.001 vs. LPS.

**Figure 9:**
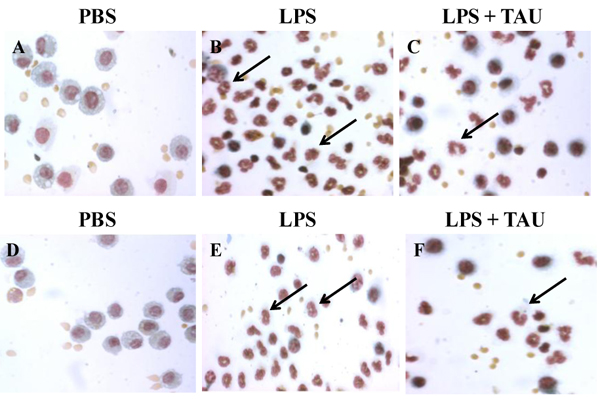
**Photomicrographs showing cells in BALF samples after a staining with Wright’s solution.** The animals received TAU (50 mg/kg/0.5 mL, i.p.) before (A-C) and after (D-F) LPS (0.02 mg). Cells from control (PBS pH 7.4) animals exhibited a normal differential count, with the majority of cells being macrophages (A and D). BALF from animals treated only with LPS (B and E) exhibited a higher number of neutrophils and only a few macrophages relative to BALF from control (PBS pH 7.4) animals. A 3-day treatment with TAU, either before (C) or after (F) LPS, reduced the number of neutrophils relative to BALF from animals receiving only LPS (magnification of 400x).

As shown in Figure 6, an acute exposure to LPS led to a profound increase in the total number of leukocytes infiltrating the lung (by 12.5-fold, P<0.001) relative to the number observed in control samples. From the results presented in Figure 7, it is apparent that the administration of TAU, either before or after LPS, led to a small reduction in the number of neutrophils that entered the lung as a result of an exposure to LPS (by 15%, P<0.05, and 11%, respectively). Moreover, as shown in Figures 8 and 9, it is evident that a treatment with TAU resulted in a significant attenuation of the enhancing action of LPS on the total number of lung neutrophils, with a pretreatment providing a greater effect (85% decrease at P<0.001 vs. LPS) than a post-treatment (53% decrease at P<0.01 vs. LPS).

### Effects of LPS and TAU-LPS on the expression of TNFR1 on BALF macrophages

**Figure 10:**
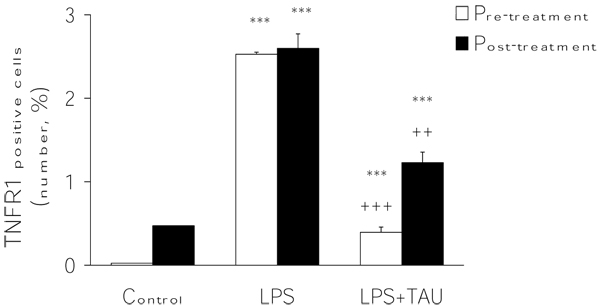
**TAU attenuated the LPS-induced increase in TNFR1-positive macrophages into BALF when given before or after LPS.** Each bar represents the mean ± S.E.M. for n = 6. ***P<0.001 vs. control; ++P<0.01 vs. LPS.

**Figure 11:**
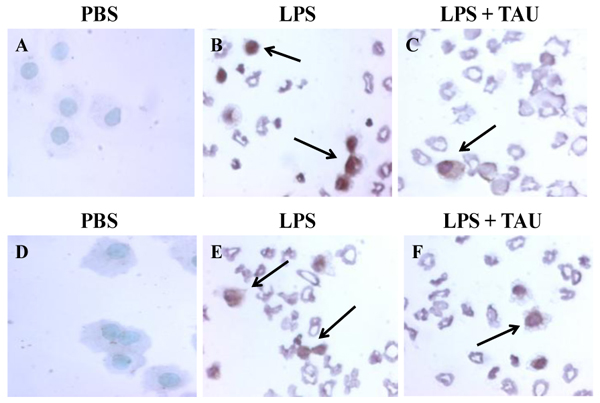
**Photomicrographs of cells from BALF samples stained with Vectastain® Elite ABC kit**. The animals received TAU (50 mg/kg/0.5 mL, i.p.) before (A-C) and after (D-F) LPS (0.02 mg). Cells from control (PBS pH 7.4) animals lacked TNFR1 positive cells (A and D). BALF from animals treated only with LPS (B and E) exhibited a modest increase in the number of TNFR1 positive macrophages relative to BALF from control (PBS pH 7.4) animals. A 3-day treatment with TAU, either before (C) or after (F) LPS, reduced the number of TNFR1 positive macrophages relative to BALF from animals receiving only LPS (magnification of 400x).

An acute exposure to LPS led to a marked increase in the expression of TNFR1 by BALF macrophages (by ~47-fold, P<0.001 vs. control). This change was significantly attenuated by a 3-day treatment with TAU, with the attenuation being greater when TAU was given ahead rather than after LPS (reductions equal to 59% and 39% respectively, P<0.01 for both vs. LPS alone) (Figures 10 and 11).

### Effects of LPS and TAU-LPS on apoptosis of lung cells

**Figure 12:**
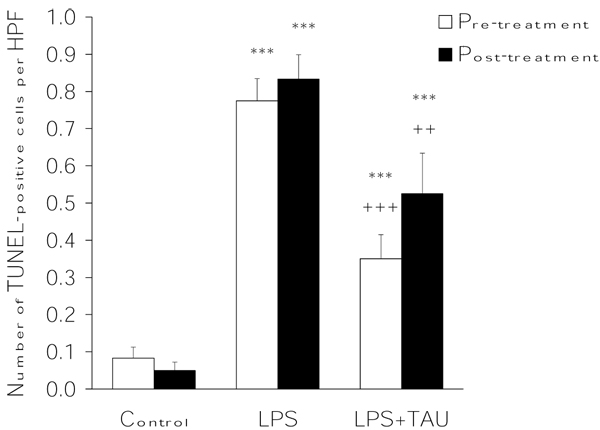
**TAU attenuated the LPS-induced increase in lung TUNEL-positive apoptotic cells when given before or after LPS.** Each bar represents the mean ± S.E.M. for n = 6. ***P<0.001 vs. control; ^++^P<0.01 and ^+++^P<0.001 vs. LPS.

**Figure 13:**
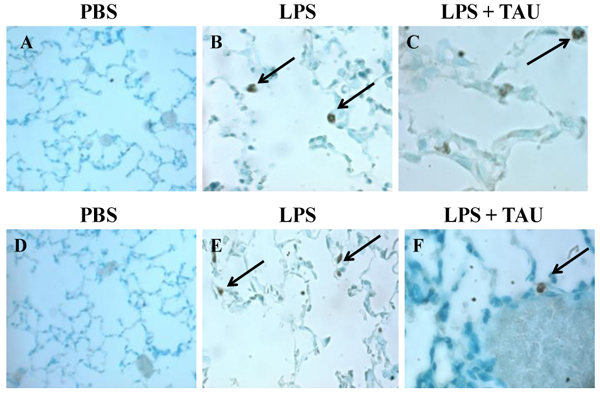
Photomicrographs of lung sections stained with ApoTag® Plus Peroxidase In Situ Apoptosis Detection kit. The animals received TAU (50 mg/kg/0.5 mL, i.p.) before (A-C) and after (D-F) LPS (0.02 mg). Sections from control (PBS pH 7.4) animals showed no TUNEL positive apoptotic cells (A and D). Sections from animals treated only with LPS (B and E) showed a modest increase in the number of TUNEL positive lung cells relative to samples from control (PBS pH 7.4) animals. A 3-day treatment with TAU, either before (C) or after (F) LPS, reduced the number of TUNEL positive lung cells relative to samples from animals receiving only LPS (magnification of 400x).

A 3-day treatment with TAU, either before or after one with LPS, resulted in a significant decrease in the number of alveolar cells that had entered apoptosis as a result of an exposure to LPS (Figures 12 and 13). Based on the results of a TUNEL assay, it was determined that a pretreatment with TAU was more effective in curtailing apoptosis (0.4 labeled cells per HPF, P<0.001) than a posttreatment (0.5 labeled cells per HPF, P<0.01) when compared to the number of apoptotic cells seen with LPS alone (0.8 labeled cells per HPF, P<0.001 vs. control). Because of the limitations imposed by the procedure used to stain the alveolar cells, only those cells occupying the septa were counted.

### Effects of LPS and TAU-LPS on the inflammatory index and on lung histology

**Figure 14:**
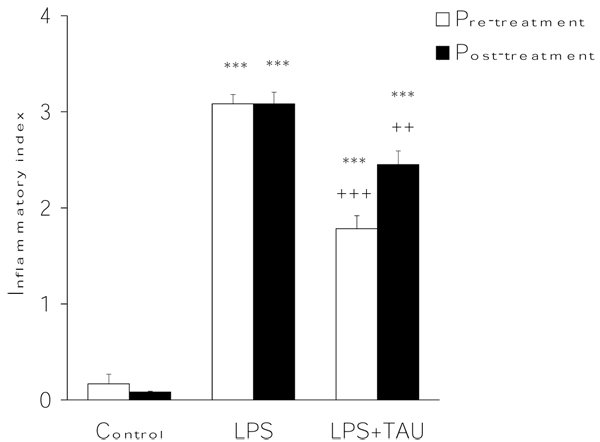
**TAU attenuated the LPS-induced lung increase in inflammatory index when given before or after LPS.** Each bar represents the mean ± S.E.M. for n = 6. ***P<0.001 vs. control; ^+++^P<0.001 and ^++^P<0.01 vs. LPS.

**Figure 15:**
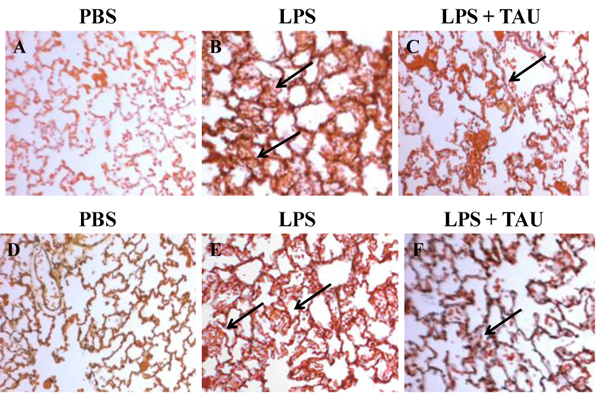
**Photomicrographs of lung sections stained with H&E.** (A-C) and (D-F) show the results for The animals received TAU (50 mg/kg/0.5 mL, i.p.) before (A-C) and after (D-F) LPS (0.02 mg). Sections from control (PBS pH 7.4) animals showed no signs of inflammation in the alveolar wall (A and D). Sections from animals treated only with LPS (B and E) showed a moderate inflammatory reaction and a mild thickening of the alveolar wall. A 3-day treatment with TAU, either before (C) or after (F) LPS, reduced the inflammatory reaction in the alveolar wall and thickening of the interstitium relative to lung sections from animals treated with LPS alone (magnification of 400x).

The inflammatory index was used to quantitatively assess the extent of lung inflammation as a result of an acute exposure to LPS. The results presented in Figure 14 suggest that the increase in the value of this parameter by LPS could be significantly attenuated by a pre- or post-treatment with TAU (from 3.0 to 1.8, P<0.001 or from 3.0 to 2.4, P<0.01. respectively). The mean inflammatory index of animals receiving only PBS (0.2) probably reflects a transient inflammatory response caused by the intratracheal instillation procedure itself. Furthermore, TAU was found to prevent the inflammatory response to LPS from spreading beyond the parenchymal tissue and into the airways (Figure 15).

## Discussion

Inflammation and oxidative stress are two closely related events that contribute to ALI as a result of an exposure to LPS. The inflammatory response that follows the instillation of LPS into the lungs appears to develop through an early and late phase process [38]. In the early phase, there is an increase in BALF neutrophils, albumin, free radical generation by the pulmonary endothelium and neutrophils, upregulation of adhesion molecules, and the release of cytokines and chemokines for the massive recruitment of macrophages and neutrophils within the pulmonary capillaries and of neutrophils in the air spaces of the lungs [14,39]. The late phase, taking place 24-48 hr after LPS instillation, is characterized by normalization of cytokine levels and increases in the number of BALF neutrophils, monocytes, macrophages and lymphocytes [38]. In the lung epithelium, TNFR1 seems to facilitate the recruitment of neutrophils after an exposure to LPS, in part by enhancing chemokine secretion [39] and to participate in a caspase-mediated signaling mechanism leading to apoptotic cell death [40,41]. On the other hand, the activation of monocytes, macrophages and other cells is the result of an interaction between LPS, bound to a LPS-binding protein (LBP) in the circulation, and CD14/TLR4 receptor complex on the target cells and culminates in the activation of transcription factors for cytokine production and ROS generation [38,42]. The upregulated release of ROS by phagocytic cells, along with proinflammatory cytokines, proteolytic enzymes and prostaglandins, eventually overwhelms the protective intracellular antioxidant mechanisms present in lung tissue and induces a state of oxidative stress characterized by the peroxidative degradation of membrane phospholipids [9,11], the inactivation of antioxidant enzymes [11,43], and the depletion of thiol-bearing molecules such as proteins [44] and GSH [13,43]. Together, these alterations will contribute to lung tissue injury manifested by epithelial permeability changes, disruption of the alveolar/epithelial barrier, and the development of interstitial edema [9,39,45].

In the present investigation, the intratracheal instillation of LPS into the lungs of Golden Syrian hamsters was sufficient to induce an oxidative stress manifested by increased formation of MDA, decreased GSH, and altered activities of CAT, GPx and SOD. The reduction in GSH, the major nonprotein sulfhydryl compound in the lung, may have been caused by direct oxidation by ROS, by its increased conversion to the oxidized (disulfide) form during the removal of hydrogen peroxide and hydroperoxides by GPx, or because of the inhibitory action of oxidants and proinflammatory mediators on γ-glutamylcysteine synthetase (γ-GCS), the key enzyme for GSH synthesis [14]. These alterations are in agreement with those reported earlier by this and other laboratories for the prooxidant action of LPS on the brain [46], heart [47], and lung [13,24,48], and support the use of antioxidants as adjuncts to the management of sepsis by Gram negative bacteria with conventional therapeutic agents.

Since a pretreatment with TAU reduced the formation of MDA significantly and returned the GSH levels to nearly control values confirm earlier results indicating that this amino acid can attenuate LPO and preserve the stores of GSH during periods of oxidative stress, including chronic ethanol consumption [49], diabetes mellitus [50,51], chemically-induced colitis [52] and iron overload [53]. Since the protective effects of TAU were greater when given as pretreatment than as a post-treatment to LPS, it is possible that TAU could be negatively influencing the formation of ROS by phagocytes and surrounding lung cells [43,52]. Alternatively, TAU could be preserving the intracellular GSH content by reducing the deleterious effect of oxidants and proinflammatory agents on redox-sensitive transcription factors regulating the gene expression of γ-GCS in the lungs [14].

CAT, GPx and SOD are the most important enzymatic defenses available to the lung for the maintenance of a normal antioxidant-oxidant balance. As previously observed with BALF samples from the lung of hamsters exposed to LPS, the activities of both CAT and SOD were reduced and that of GPx increased [13]. Although the same trend of results has been described earlier for the liver of rodents acutely treated with LPS [16,54], considerable variability seems to exist among laboratories regarding the effect of this endotoxin on the lung activities of antioxidant enzymes. For example, it has been reported that LPS reduced the activity of CAT, GPx and SOD in the lung of mice inhaling a solution of LPS in physiological saline for 5 days within an inhalation chamber [11]; had no effect on the GPx activity [55], and increased the CAT activity while decreasing that of SOD activity [25] in the lung. Some of the factors underlining these discrepancies among antioxidant enzyme activities during an inflammatory response to LPS preceding ALI could be the animal species used in the experiments [56,57] and factors related to the endotoxin itself such as the route of administration [56], the dose administered [56], the duration of the exposure [11], and the concentration of the solution used [56].

The higher than normal number of total leukocytes and neutrophils found here for BALF samples from hamsters instilled with LPS confirms the role of endotoxemia as a stimulus for the migration of neutrophils into the lung in response to the in vivo production of chemotatic factor by activated alveolar neutrophils and macrophages [58]. The inhibitory action of TAU on LPS-induced infiltration of the lung by leukocytes in general and by neutrophils in particular appears to occur subsequent to its conversion to TAU chloramine (TAU-Cl) in activated neutrophils upon interaction with hypochlorous acid (HClO) generated in the presence of the myeloperoxidase (MPO)-halide system during the phagocytosis of bacteria [30,59]. While TAU-Cl has been reported to downregulate the production of inflammatory mediators such as NO, prostaglandin E_2_ and tumor necrosis factorα- (TNF-α) in activated macrophages and neutrophils in an autocrine manner [60], and to suppress superoxide anion and IL-6 and IL-8 production in activated neutrophils [61,62], TAU itself was unable to suppress proinflammatory cytokine production [63]. Similarly, although TAU and TAU-Cl can both reduce the formation of products of the respiratory burst by interferon-γ (IFN-γ)-stimulated peritoneal neutrophils, the effect of TAU-Cl is achieved at a much lower dose that that required for TAU [61]. A similar difference in effects between TAU and TAU-Cl has been verified in vitro for eosinophils [64]. In addition to its effect on the influx of both leukocytes and neutrophils into the lung airspaces, TAU was also found here to attenuate the expression of TNFR1 on macrophages present in BALF as a result of an exposure to LPS, again to a greater extent when given before than after LPS. These findings lend support the long held view that TAU can serve as a potent inhibitor of inflammation and immune response in the lung [65,66], possibly by modulating the transcriptionally regulated production of proinflammatory and chemoattracting cytokines by alveolar macrophages [59,62] and other activated leukocytes [67,68] for the recruitment of blood monocytes and neutrophils into the lung. In this context, TAU-Cl appears to influence cytokine release by acting as a negative effector on the signal pathway for the nuclear translocation and activation of NF-κB for cytokine synthesis by neutrophils, most likely by oxidizing Met45 residue of IκBα [69]. An additional consequence of such a modulatory effect by TAU-Cl, and of relevance to the proinflammatory action of LPS in the lung, is the decreased production of MCP-1 and MCP-2, two chemokines that participate in the recruitment of macrophages by activated neutrophils [59]. In addition, TAU-Cl may be protecting the lungs by preventing the transendothelial migration of neutrophils by shortening their rolling velocity [70]; and by reducing lung arterial pressure, hypoxia, MPO activity, and the excessive release of inflammatory mediators and of products of the respiratory burst activity by neutrophils [30].

As one of the major factors for ALI, LPS is found to induce disseminated endothelial apoptosis prior to endothelial tissue damage and that caspases play an important role in the process [12]. While the protective actions of TAU as TAU-Cl in the lung contrast sharply with the known proapoptotic action associated with this chlorinated TAU derivative on certain cells as a result of direct damage to the mitochondrion [71], in the present study TAU was found to reduce lung cell apoptosis. Such a protection might have resulted from a decrease in TNFR1 expression on lung cells, needed for stimulation of the TNF-induced signaling pathway of apoptosis, and from inhibition of a key caspase downstream in the pathway, which was previously shown by this laboratory to include caspase-3 [31].

## Conclusions

The data reported here strongly suggest that TAU can act in the lung as a protectant against the proinflammatory, prooxidant and apoptotic actions of bacterial endotoxin, and that such a protection is achievable regardless of whether TAU is administered before or after the endotoxin. The magnitude of the protective actions of TAU in the lung against endotoxin can be readily assessed by monitoring the changes in BALF cell counts, TNFR1 expression, the occurrence of alveolar apoptosis, and in indices of oxidative stress; and. are in close agreement with the results gathered by histopathological examination of lung tissue samples.

## Abbreviations

H_2_O_2_: hydrogen peroxide; LPS: lipopolysaccharide; PBS: phosphate buffered saline; GSH: reduced glutathione; TBARS: thiobarbituric acid reactive substances; TCA: trichloroacetic acid; SSA: sulfosalicylic acid; TEP: 1,1,3,3-tetraethoxypropane; TBA: thiobarbituric acid; HCl: hydrochloric acid; BALF: bronchoalveolar lavage fluid; MDA: malondialdehyde; CAT: catalase; SOD: superoxide dismutase; GPx: glutathione peroxidase; DTNB: 5,5’-dithiobis(2-nitrobenzoic acid); NBT: nitroblue tetrazolium; EDTA: ethylenediaminetetraacetic acid; H: hematoxylin; E: eosin.

## Competing interests

The authors declare that they have no competing interests.

## Authors’ contributions

TMB carried out all experimental work on live animals, performed the cytological and histopathological evaluations. In addition, prepared the figures, performed the statistical analyses, helped with the collection of bibliographical information, and made editorial comments to the article. SNP performed all the biochemical assays on the lung samples, and helped with the preparation of the figures. CAL conceived the project and guided its development, assembled, organized and interpreted the experimental data, and reviewed the pertinent scientific literature.
